# Feasibility of Multi-Wavelength LED Irradiation on the Skull and Preliminary Glycemic Outcomes in Individuals with Type 2 Diabetes: A Prospective Pilot Study

**DOI:** 10.3390/life16091411

**Published:** 2026-08-25

**Authors:** Chi-Chang Wu, Jung-Sheng Chiang, Chang-Yin Lee, Chih-Yu Wang

**Affiliations:** 1 Department of Electrical Engineering, I-Shou University, Kaohsiung 84001, Taiwan; isu11301002d@cloud.isu.edu.tw (C.-C.W.); cjs@isu.edu.tw (J.-S.C.); 2 Department of Chinese Medicine, School of Chinese Medicine for Post Baccalaureate, E-DA Hospital, I-Shou University, Kaohsiung 82445, Taiwan; 3 Department of Biomedical Engineering, I-Shou University, Kaohsiung 82445, Taiwan

**Keywords:** photobiomodulation, LED irradiation, skull, type 2 diabetes mellitus, glycemic outcomes, prospective pilot study

## Abstract

Background: Type 2 diabetes mellitus (T2DM) is a chronic metabolic disorder characterized by impaired glucose homeostasis, insulin resistance, and progressive β-cell dysfunction. Increasing evidence suggests that glucose homeostasis is regulated not only by peripheral metabolic organs but also by central nervous system and autonomic pathways. Photobiomodulation (PBM) has been reported to modulate mitochondrial function, oxidative stress, vascular responses, and neural activity; however, its potential influence on glycemic outcomes remains largely unexplored. Objective: This prospective pilot study aimed to evaluate the feasibility of applying multi-wavelength LED irradiation to a predefined cranial region and to explore preliminary glycemic outcomes in individuals with T2DM. Methods: Seven participants with physician-diagnosed T2DM receiving stable antidiabetic medication regimens were enrolled. Multi-wavelength LED irradiation (660, 850, and 940 nm) was applied bilaterally to the predefined S3 region of the skull. Irradiation parameters included an irradiance of 5 mW/cm^2^ (0.005 W/cm^2^), an energy density of 4.5 J/cm^2^, and an exposure duration of 900 s per session. Treatments were administered three times weekly for four weeks (12 sessions). Primary outcome measures included fasting blood glucose (Glucose AC) and glycated hemoglobin (HbA1c). Secondary outcomes included mean blood glucose, fasting insulin, C-peptide, homeostasis model assessment of insulin resistance (HOMA-IR), lipid profile, and C-reactive protein (CRP). Exploratory paired statistical analyses were performed following assessment of data normality using the Shapiro–Wilk test. Results: Following the four-week intervention, fasting blood glucose and mean blood glucose were significantly reduced (*p* = 0.026 and *p* = 0.044, respectively), whereas HbA1c demonstrated a borderline reduction (*p* = 0.051). Changes in fasting insulin, C-peptide, HOMA-IR, lipid profile, and CRP were variable and did not reach statistical significance. No intervention-related adverse events were observed, and all participants completed the study protocol. Conclusions: Multi-wavelength LED irradiation applied to the predefined S3 region of the skull was feasible, safe, and well tolerated in individuals with T2DM. The observed preliminary glycemic outcomes support the feasibility of further investigation in larger randomized controlled trials. However, because of the small sample size, absence of a control group, and exploratory study design, the present findings should be interpreted cautiously and should not be considered evidence of clinical efficacy or causal effects.

## 1. Introduction

Type 2 diabetes mellitus (T2DM) is one of the most prevalent chronic metabolic diseases worldwide and remains a major public health challenge. Persistent hyperglycemia is associated with progressive vascular injury, oxidative stress, chronic inflammation, and dysfunction of multiple organ systems, leading to increased morbidity, mortality, and healthcare costs [1]. Although pharmacological therapy and lifestyle modification remain the cornerstones of diabetes management, many individuals fail to achieve satisfactory glycemic control, highlighting the need to explore complementary non-pharmacological interventions [1].

Growing evidence indicates that glucose homeostasis is regulated not only by peripheral metabolic organs but also by the central nervous system (CNS) through integrated neuroendocrine and autonomic mechanisms [2,3,4,5,6,7,8,9]. The hypothalamus and brainstem coordinate hormonal, nutritional, and neural signals to regulate hepatic glucose production, pancreatic insulin secretion, peripheral glucose utilization, and overall energy metabolism [2,3,4,5,6,7]. Dysregulation of these neuro-metabolic pathways has been implicated in insulin resistance, obesity, metabolic syndrome, and T2DM [5,6,7,8,9]. These findings suggest that interventions capable of modulating central neural function may also influence systemic metabolic regulation.

Photobiomodulation (PBM) is a non-invasive technique that utilizes red and near-infrared light to induce biological responses through mitochondrial photoreceptors, particularly cytochrome c oxidase, resulting in modulation of cellular metabolism, inflammation, oxidative stress, and microcirculation [10,11,12,13]. Recent studies have further demonstrated that cranial PBM may influence cerebral blood flow, neuronal activity, mitochondrial function, and functional brain connectivity, suggesting its potential as a non-invasive neuromodulation approach [13,14,15,16,17,18,19,20,21]. Nevertheless, direct clinical evidence investigating whether cranial PBM influences glycemic outcomes in individuals with T2DM remains limited.

In the present study, PBM was applied to a predefined cranial region referred to as the S3 area, which was selected according to the craniosacral therapy framework proposed by Cheng The S3 area is defined as the intersection between a palpable transverse fascial tension ridge and the sagittal suture in the posterior parietal region, providing a reproducible external anatomical landmark for irradiation A multi-wavelength LED device incorporating 660, 850, and 940 nm light was employed. The 660 nm wavelength has been widely used because of its interaction with cytochrome c oxidase, whereas the 850 and 940 nm wavelengths provide greater tissue penetration. Rather than evaluating the biological effects of individual wavelengths, simultaneous application of these three wavelengths was intended to provide complementary optical characteristics across superficial and deeper tissues [10,11,12,13,14,15,16,17].

Therefore, the objective of this prospective pilot study was to evaluate the feasibility of applying multi-wavelength LED irradiation to the predefined S3 region of the skull and to explore preliminary glycemic outcomes in individuals with T2DM. Given the exploratory design, limited sample size, and absence of a control group, this study was intended to generate preliminary clinical observations to inform the design of future randomized controlled trials rather than to establish clinical efficacy or causal mechanisms.

## 2. Materials and Methods

### 2.1. Study Design

This study was designed as a prospective pilot clinical study to evaluate the feasibility of applying multi-wavelength LED irradiation to a predefined cranial region in individuals with type 2 diabetes mellitus (T2DM) and to explore preliminary glycemic outcomes following a standardized irradiation protocol. Owing to the exploratory nature of the study, the primary objective was to assess the feasibility, safety, and preliminary clinical observations associated with the intervention rather than to establish clinical efficacy or causal relationships.

The intervention was conducted over a four-week period. Participants received multi-wavelength LED irradiation three times per week, resulting in a total of 12 treatment sessions. Clinical and laboratory assessments were performed at two predefined time points: immediately before the first treatment session (Pre) and immediately after completion of the four-week intervention (Post). All participants completed the intervention and follow-up assessments according to the study protocol.

To minimize potential confounding, participants continued their routine medical care throughout the study. Antidiabetic medications and other regular treatments remained unchanged during the intervention period, and no additional glucose-lowering interventions were introduced. Because the primary purpose of this pilot study was to evaluate the feasibility of a standardized multi-wavelength LED irradiation protocol under routine clinical conditions, no sham-treated or untreated control group was included. Consequently, the findings should be interpreted as exploratory observations and should not be regarded as evidence of treatment efficacy.

For four participants, historical laboratory records obtained during routine clinical follow-up before study enrollment were available. These records confirmed that antidiabetic medication regimens had remained stable before enrollment and provided longitudinal HbA1c measurements for clinical reference. The historical data were reviewed to provide additional clinical context but were not included in the formal statistical analyses.

The primary outcome measures were fasting blood glucose (Glucose AC) and glycated hemoglobin (HbA1c). Secondary outcome measures included mean blood glucose, fasting insulin, C-peptide, homeostasis model assessment of insulin resistance (HOMA-IR), lipid profile, and C-reactive protein (CRP). Statistical analyses of paired pre- and post-measurements are described in Section 2.8.

The study was conducted in accordance with the CARE (CAse REport) guidelines for clinical case reporting [22] and the ethical principles of the Declaration of Helsinki [23]. Ethical approval and written informed consent procedures are described in Section 2.3.

### 2.2. Participants

Seven Taiwanese adults with physician-diagnosed type 2 diabetes mellitus (T2DM) were prospectively recruited from a home nursing service in southern Taiwan using convenience sampling. All participants were receiving routine clinical care before enrollment. Baseline demographic and clinical characteristics are summarized in Table 1. The study cohort consisted of four males and three females, with a mean age of 70.3 ± 11.4 years (range: 53–82 years). All participants had concomitant hypertension and dyslipidemia and were receiving stable antidiabetic medication regimens before enrollment. No changes in antidiabetic medications or other routine medical treatments were made throughout the intervention period, thereby minimizing potential confounding attributable to medication adjustment.

Participants were eligible if they met all of the following criteria:

(1) age ≥ 20 years;
(2) physician-diagnosed T2DM for at least six months;
(3) HbA1c ≥ 6.5% according to the American Diabetes Association (ADA) diagnostic criteria [24];
(4) stable antidiabetic medication regimen before enrollment and throughout the study period;
(5) presence of an identifiable S3 fascial tension ridge upon standardized clinical palpation; and
(6) willingness to participate and provide written informed consent.

Participants were excluded if they met any of the following criteria:

(1) photosensitivity disorders or known hypersensitivity to light exposure;
(2) endocrine disorders other than T2DM that could substantially affect glucose metabolism;
(3) initiation of insulin therapy or major changes in antidiabetic medications within three months before enrollment;
(4) previous cranial or spinal surgery;
(5) severe neurological disorders or neurodegenerative diseases;
(6) severe cardiovascular disease or other unstable medical conditions; or
(7) inability to comply with the study protocol.

Because this investigation was designed as a prospective pilot clinical study, only participants who satisfied all eligibility criteria were enrolled. All enrolled participants completed the four-week intervention and post-intervention assessments according to the study protocol. Consequently, no participants withdrew or were excluded from the final analysis, and all seven participants were included in the statistical analyses.

### 2.3. Ethical Considerations

The study protocol was reviewed and approved by the Institutional Review Board (IRB) of I-Shou University, Kaohsiung, Taiwan (Approval No. EMRP-114-150) before participant recruitment. The study was conducted in accordance with the ethical principles of the Declaration of Helsinki and its subsequent amendments [23].

Before enrollment, all participants received a detailed explanation of the study objectives, study procedures, potential benefits, possible risks, and their right to withdraw from the study at any time without affecting their routine medical care. Written informed consent was obtained from all participants before any study-related procedures were performed.

Participant confidentiality and privacy were strictly protected throughout the study. Personal identifying information was removed from the research dataset before analysis, and all study data were anonymized and analyzed in aggregate. Only members of the research team had access to the study data.

Because this study involved a non-invasive photobiomodulation intervention using low-power LED irradiation, no intervention-related adverse events were observed during the study period. Participants were monitored throughout each treatment session, and appropriate procedures were in place to discontinue the intervention if any unexpected discomfort or adverse event occurred.

### 2.4. Intervention Device

A custom-designed multi-wavelength light-emitting diode (LED) photobiomodulation (PBM) device was developed specifically for this prospective pilot study. The device was designed for research purposes and was not a commercially available medical device. Each LED module incorporated three integrated LED chips emitting wavelengths of 660 nm (red), 850 nm (near-infrared), and 940 nm (near-infrared), enabling simultaneous multi-wavelength irradiation. Two identical handheld LED probes, each containing one integrated multi-wavelength LED module, were used to deliver bilateral irradiation to the predefined S3 region of the skull.

The irradiation parameters were standardized and remained identical for all participants throughout the study. Each LED module emitted an optical output power of 5 mW, corresponding to an irradiance of 5 mW/cm^2^ (0.005 W/cm^2^) under the standardized irradiation conditions used in this study. The predetermined energy density (fluence) was 4.5 J/cm^2^, achieved by an exposure duration of 900 s (15 min). Irradiation was delivered in a non-contact manner, with the LED probes positioned approximately 0.5–1.0 cm above the scalp surface throughout each treatment session. All irradiation parameters are summarized in Table 2.

The three wavelengths were selected to provide complementary optical characteristics for PBM. The 660 nm wavelength has been extensively investigated because of its interaction with cytochrome c oxidase, a principal mitochondrial photoacceptor involved in cellular bioenergetics and photobiomodulation [10,11,12,13]. In contrast, the 850 nm and 940 nm near-infrared wavelengths exhibit greater tissue penetration than visible red light and may deliver light energy to deeper biological tissues [14,15,16,17]. Rather than evaluating the biological effects of individual wavelengths, the present study employed simultaneous multi-wavelength irradiation to provide complementary optical characteristics within a standardized treatment protocol.

The irradiation duration of 900 s was predetermined to achieve the target energy density of 4.5 J/cm^2^ while maintaining a constant irradiance of 5 mW/cm^2^. The same device, identical irradiation parameters, and standardized operating procedures were used throughout the study without modification, thereby ensuring treatment consistency among all participants.

### 2.5. Definition of the S3 Area

The S3 area used in this study was defined as an operational anatomical landmark for irradiation and should not be interpreted as a recognized anatomical structure or an established cranial acupoint. The operational definition was adopted from the craniosacral therapy concept proposed by Cheng [25] and was used exclusively to standardize the irradiation location in this prospective pilot study.

According to this operational definition, the S3 area is located over the posterior parietal region along the sagittal suture, approximately three finger-widths anterior to the lambda (the junction of the sagittal and lambdoid sutures). Within this region, a palpable transverse fascial tension ridge crossing the sagittal suture is identified by standardized manual palpation, and the intersection between the ridge and the sagittal suture is designated as the S3 point. The surrounding region centered on this point was defined as the S3 irradiation area throughout the study [25].

Localization of the S3 area was performed by the same investigator using a standardized palpation procedure before every treatment session to ensure consistent placement of the LED probes. The bilateral LED probes were positioned symmetrically over the predefined S3 area in a non-contact manner while maintaining a fixed irradiation distance of approximately 0.5–1.0 cm, as described in Section 2.4. The irradiation location and procedure were kept identical for all participants throughout the study to minimize inter-session variability.

Because the S3 area represents an operational landmark derived from a craniosacral therapy concept rather than a universally accepted anatomical structure, the present study does not claim anatomical or physiological validation of the S3 concept. Instead, the predefined S3 area was used solely as a standardized and reproducible irradiation site to evaluate the feasibility and preliminary glycemic outcomes of multi-wavelength LED photobiomodulation in individuals with type 2 diabetes mellitus.

### 2.6. Multi-Wavelength LED Irradiation Protocol

Participants received multi-wavelength LED photobiomodulation (PBM) three times per week for four consecutive weeks, resulting in a total of 12 irradiation sessions. Each treatment session lasted 900 s (15 min), using the irradiation parameters summarized in Table 2.

Before each irradiation session, participants rested in a seated position for 10 min to minimize the potential influence of recent physical activity on physiological status. The S3 area was then identified by the same experienced operator using the standardized palpation procedure described in Section 2.5 [25]. Two handheld LED probes were positioned bilaterally over the predefined S3 areas in a non-contact manner, maintaining a distance of approximately 0.5–1.0 cm above the scalp surface throughout the irradiation period.

All irradiation sessions were conducted in the same treatment room under controlled environmental conditions, with a room temperature of 25 ± 2 °C and a relative humidity of 55–60%, as summarized in Table 2.

To minimize potential confounding factors, participants were instructed to maintain their usual lifestyle, dietary habits, and prescribed medications throughout the study. No changes in antidiabetic medications or other routine medical treatments were made during the intervention period.

All irradiation sessions were performed by the same operator using the same LED device, identical irradiation parameters, and a standardized operating procedure to ensure consistency throughout the study.

Participants were monitored for any discomfort or adverse events during and immediately after each irradiation session. No intervention-related adverse events were observed throughout the study.

### 2.7. Outcome Measurements

The predefined outcome measures were categorized as primary and secondary outcomes before initiation of the study. All laboratory assessments were performed at two predefined time points: immediately before the first irradiation session (Pre) and immediately after completion of the four-week intervention (Post). Venous blood samples were collected after an overnight fast and analyzed by a certified clinical laboratory using standardized analytical procedures. Laboratory results were summarized using descriptive statistics as appropriate.

The primary outcome measures were fasting blood glucose (Glucose AC) and glycated hemoglobin (HbA1c), which are widely accepted clinical indicators of glycemic control in individuals with type 2 diabetes mellitus (T2DM) [24]. Fasting blood glucose was included because it reflects short-term glycemic status and is responsive to changes occurring during relatively short intervention periods, whereas HbA1c was included to evaluate longer-term glycemic control. Because HbA1c reflects the average blood glucose level over approximately two to three months, changes observed after the four-week intervention were interpreted cautiously.

The secondary outcome measures included mean blood glucose, fasting insulin, C-peptide, homeostasis model assessment of insulin resistance (HOMA-IR), lipid profile (total cholesterol, triglycerides, HDL-cholesterol, LDL-cholesterol, total cholesterol/HDL ratio, and LDL/HDL ratio), and C-reactive protein (CRP). These variables were selected to provide a broader assessment of glucose metabolism, insulin secretion, insulin resistance, lipid metabolism, and systemic inflammatory status associated with T2DM.

### 2.8. Statistical Analysis

Statistical analyses were performed using IBM SPSS Statistics for Windows, Version 27.0 (IBM Corp., Armonk, NY, USA). Owing to the exploratory nature of this prospective pilot case series and the small sample size (n = 7), all inferential analyses were considered exploratory and were intended to provide preliminary estimates of pre–post changes rather than confirmatory evidence of efficacy.

The normality of paired differences was assessed using the Shapiro–Wilk test. Variables with normally distributed paired differences were analyzed using the paired-samples *t*-test, whereas variables that did not meet the normality assumption were analyzed using the Wilcoxon signed-rank test. Normally distributed variables are presented as mean ± standard deviation (SD), whereas non-normally distributed variables are presented as median (Q1, Q3).

For variables analyzed using the paired-samples *t*-test, the mean difference (Post−Pre) and its 95% confidence interval (95% CI) were calculated. Effect sizes were expressed as Cohen’s d. For variables analyzed using the Wilcoxon signed-rank test, the corresponding effect size was expressed as the rank-biserial correlation.

All statistical tests were two-tailed, and a *p* value < 0.05 was considered statistically significant. Given the exploratory nature and limited sample size of the study, *p* values were interpreted cautiously and were not considered confirmatory evidence of efficacy. Historical laboratory records available for four participants were reviewed only to provide additional longitudinal clinical context and were not included in the formal statistical analyses.

## 3. Results

### Changes in Glycemic and Metabolic Parameters

All seven participants completed the four-week multi-wavelength LED photobiomodulation (PBM) intervention and were included in the final analysis. No participants withdrew from the study, and no intervention-related adverse events, treatment interruptions, or protocol deviations were observed during the study period.

Changes in laboratory measurements between the pre- and post-intervention assessments are summarized in Table 3.

**Figure 1:**
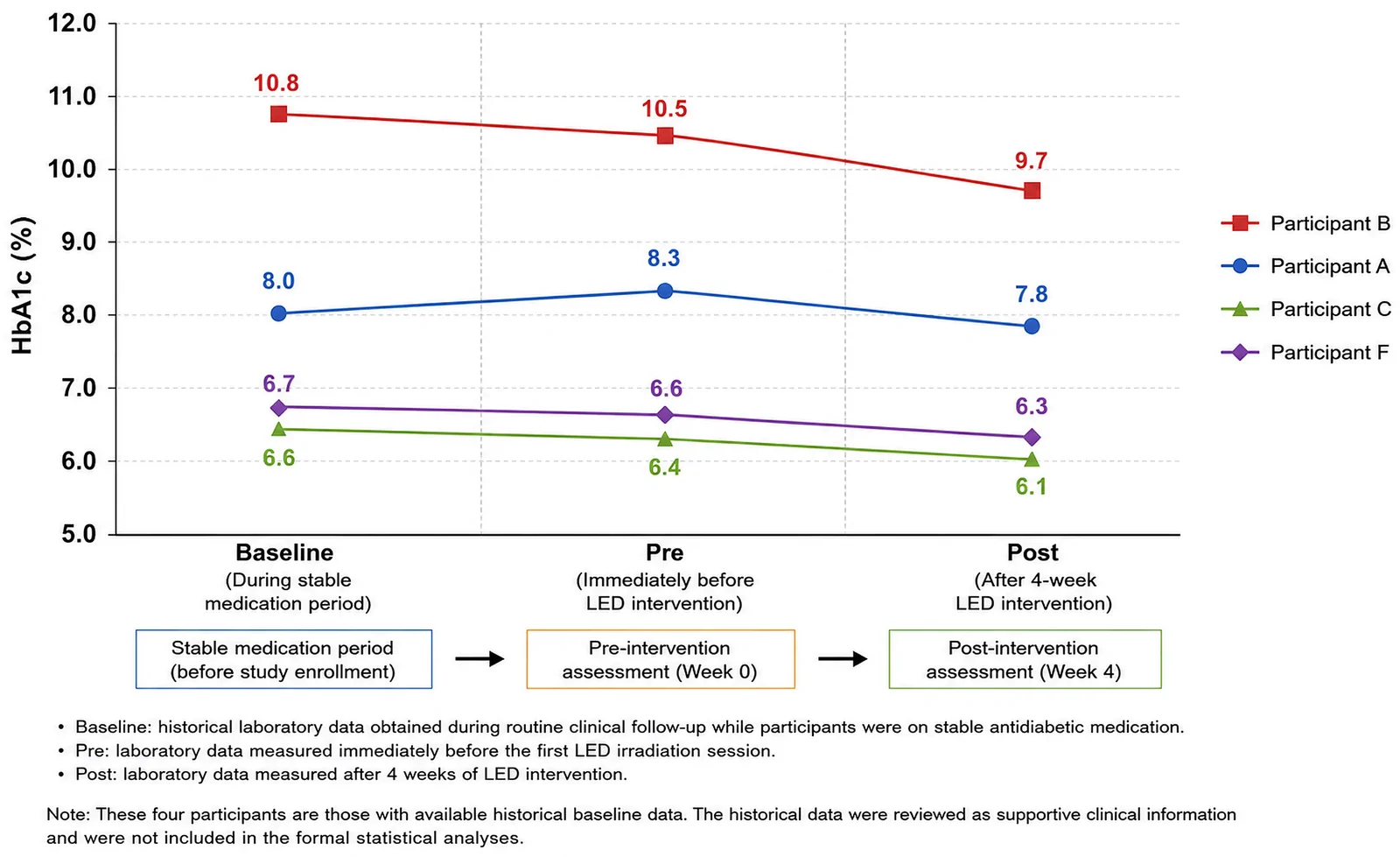
HbA1c Trends for Four Participants with Available Historical Baseline Data.

Historical laboratory records were available for four participants. Figure 1 shows the longitudinal HbA1c measurements obtained at the historical baseline, immediately before the intervention (Pre), and after the four-week intervention (Post). These historical data were not included in the formal statistical analyses and are presented descriptively to provide longitudinal clinical context; they should not be interpreted as a statistical comparison or as evidence of intervention efficacy.

Among the primary outcome measures, fasting blood glucose (Glucose AC) showed a statistically significant reduction after the intervention (*p* = 0.026). HbA1c also decreased from the pre-intervention assessment; however, the difference did not reach statistical significance (*p* = 0.051).

Among the secondary outcome measures, mean blood glucose demonstrated a statistically significant reduction after the intervention (*p* = 0.044). C-peptide, fasting insulin, HOMA-IR, lipid profile, and C-reactive protein (CRP) showed variable changes between the pre- and post-intervention assessments; however, none of these variables demonstrated statistically significant differences (all *p* > 0.05). For CRP, which showed a non-normal distribution, values increased from 1.30 (1.20, 1.43) mg/L at pre-intervention to 2.60 (1.30, 4.06) mg/L at post-intervention, but the difference was not statistically significant (Wilcoxon signed-rank test, *p* = 0.156).

## 4. Discussion

The present prospective pilot study evaluated the feasibility of applying multi-wavelength LED photobiomodulation (PBM) to a predefined cranial region in individuals with type 2 diabetes mellitus (T2DM). All seven participants completed the four-week intervention without treatment interruption or intervention-related adverse events, indicating that the standardized irradiation protocol was feasible, safe, and well tolerated under the study conditions. Exploratory analyses demonstrated significant reductions in fasting blood glucose (Glucose AC) and mean blood glucose following the intervention, whereas HbA1c showed a borderline reduction that did not reach statistical significance. In contrast, fasting insulin, C-peptide, homeostasis model assessment of insulin resistance (HOMA-IR), lipid profile, and C-reactive protein (CRP) did not demonstrate statistically significant changes. Because of the exploratory study design, small sample size, and absence of a control group, these findings should be interpreted as preliminary clinical observations rather than evidence of treatment efficacy or causal relationships.

The observed reductions in fasting blood glucose and mean blood glucose suggest that cranial PBM may influence short-term glycemic regulation. Fasting blood glucose generally responds more rapidly to physiological changes than HbA1c, whereas HbA1c reflects average glycemic status over approximately two to three months. Consequently, the borderline reduction in HbA1c observed after only four weeks of intervention is not unexpected and should be interpreted cautiously. Collectively, these findings suggest that short-term glycemic indicators may be more responsive than HbA1c for evaluating preliminary metabolic changes during brief exploratory PBM interventions.

The biological mechanisms underlying the observed glycemic changes remain uncertain. Previous studies have demonstrated that the central nervous system contributes to systemic glucose homeostasis through integrated autonomic and neuroendocrine regulation, while PBM has been reported to influence mitochondrial bioenergetics, oxidative stress, vascular function, and cellular metabolism. It is therefore biologically plausible that cranial PBM may indirectly influence glucose regulation through neurophysiological or metabolic pathways. However, the present study did not include direct assessments of autonomic nervous system activity, cerebral perfusion, neuroimaging, continuous glucose monitoring, or other mechanistic biomarkers. Consequently, no mechanistic conclusions can be drawn from the present findings, and the proposed physiological pathways remain speculative. Furthermore, because the three wavelengths (660, 850, and 940 nm) were delivered simultaneously throughout the intervention, the contribution of each individual wavelength cannot be determined. Future studies comparing single-wavelength and combined-wavelength PBM protocols are warranted to clarify their respective biological effects.

Historical laboratory records were available for four participants and provided supplementary longitudinal clinical information beyond the formal pre- and post-intervention assessments. Because these retrospective measurements were obtained during routine clinical follow-up before study enrollment, they were presented solely to provide descriptive clinical context and were intentionally excluded from the formal statistical analyses. Accordingly, the historical HbA1c trends should not be interpreted as evidence of treatment efficacy or as a substitute for a concurrent control group.

Despite its exploratory nature, this study has several strengths. First, all participants received an identical multi-wavelength PBM protocol using standardized irradiation parameters, environmental conditions, and operating procedures, thereby minimizing procedural variability. Second, antidiabetic medication regimens remained unchanged throughout the intervention period, reducing potential confounding related to medication adjustment. Third, multiple metabolic biomarkers were evaluated, allowing a broader assessment of glycemic regulation, insulin secretion, insulin resistance, lipid metabolism, and systemic inflammatory status. Finally, the inclusion of historical laboratory data for four participants provided additional longitudinal clinical context that complemented the prospective observations, although these retrospective data were not incorporated into the statistical analyses.

Several limitations should be acknowledged. First, the study included only seven participants, limiting statistical power and the generalizability of the findings. Second, the single-arm design without a sham-treated or untreated control group precludes attribution of the observed changes solely to the intervention. Third, the intervention period was limited to four weeks, preventing evaluation of longer-term glycemic outcomes, particularly changes in HbA1c. Fourth, although antidiabetic medication regimens remained stable throughout the study, concomitant medications, chronic comorbidities, the wide age range of participants, differences in baseline metabolic status, and other individual clinical characteristics may also have contributed to the observed variability and could not be adjusted because of the limited sample size. Finally, objective assessments of autonomic nervous system function, cerebral blood flow, neurophysiology, or continuous glucose monitoring were not performed, preventing direct investigation of the biological mechanisms underlying the observed glycemic changes. In addition, this exploratory study evaluated only a single irradiation protocol; therefore, potential dose-dependent or biphasic responses associated with different irradiation parameters could not be assessed.

Future randomized, sham-controlled clinical trials with larger sample sizes, longer intervention periods, and comprehensive mechanistic assessments are warranted to validate these preliminary observations. Incorporating objective physiological measurements, such as autonomic function assessment, cerebral perfusion imaging, neuroimaging, continuous glucose monitoring, and comparisons of different irradiation parameters and wavelength combinations, would further clarify the mechanisms and potential clinical applications of cranial PBM for individuals with T2DM.

## 5. Conclusions

This prospective pilot study demonstrated that multi-wavelength LED photobiomodulation (PBM) applied to a predefined cranial region was feasible, safe, and well tolerated in individuals with type 2 diabetes mellitus (T2DM). All participants successfully completed the four-week intervention without treatment interruption or intervention-related adverse events, supporting the practical feasibility of implementing the standardized irradiation protocol under the study conditions.

Exploratory analyses suggested potential improvements in selected glycemic outcomes, with statistically significant reductions observed in fasting blood glucose and mean blood glucose following the intervention, whereas changes in HbA1c and other metabolic biomarkers were limited during the four-week study period. These findings provide preliminary clinical observations that support further investigation of cranial PBM and its potential influence on glycemic outcomes in individuals with T2DM.

Nevertheless, the present findings should be interpreted with caution because of the exploratory study design, small sample size, absence of a sham-treated or untreated control group, and short intervention duration. Accordingly, the results should not be interpreted as evidence of clinical efficacy or causal relationships.

Future randomized, sham-controlled clinical trials with larger sample sizes, longer follow-up periods, and comprehensive mechanistic assessments are warranted to validate these preliminary observations. Comparative evaluation of different irradiation parameters, wavelength combinations, and objective physiological measurements will be important for clarifying the biological mechanisms and potential clinical applications of cranial PBM in T2DM.

## Figures and Tables

**Table 1:** Baseline Characteristics of the Participants (*n* = 7).

Characteristic	Value
Number of participants	7
Age, years	70.3 ± 11.4
Male sex	4 (57.1%)
Female sex	3 (42.9%)
Age range, years	53–82
Type 2 diabetes mellitus	7 (100%)
Hypertension	7 (100%)
Dyslipidemia	7 (100%)
Stable antidiabetic medication	7 (100%)
Antihypertensive medication	7 (100%)
Lipid-lowering medication	7 (100%)

**Table 2:** Irradiation Parameters of the Multi-Wavelength LED Photobiomodulation Protocol.

Parameter	Specification	Parameter	Specification
Wavelengths	660 nm, 850 nm, 940 nm	Treatment frequency	3 sessions/week
LED probes	2	Total treatment sessions	12
Irradiation site	S3 area of the skull	Application mode	Non-contact
Output power (per LED)	5 mW	Distance from scalp	0.5–1.0 cm
Irradiance (Power density)	5 mW/cm^2^ (0.005 W/cm^2^)	Room temperature	25 ± 2 °C
Energy density (Fluence)	4.5 J/cm^2^	Relative humidity	55–60%
Exposure duration	900 s (15 min)	Rest period before irradiation	10 min
LED package size	2 × 2 mm	Effective irradiation field	Illuminated area produced by each LED module

**Table 3:** Comparison of Laboratory Measurements Between the Pre- and Post-Intervention Assessments (n = 7).

Laboratory Parameter	Pre	Post	Mean Difference (95% CI)	Statistical Test	Effect Size	*p* Value
Glucose AC (mg/dL)	165.00 ± 60.89	131.29 ± 37.62	−33.71 (−61.75 to −5.67)	Paired *t*-test	Cohen’s d = −1.11	0.026
HbA1c (%)	7.43 ± 1.49	7.09 ± 1.30	−0.34 (−0.69 to 0.00)	Paired *t*-test	Cohen’s d = −0.92	0.051
Mean blood glucose (mg/dL)	166.71 ± 42.99	156.57 ± 37.50	−10.14 (−19.91 to −0.38)	Paired *t*-test	Cohen’s d = −0.96	0.044
C-peptide (ng/mL)	3.96 ± 2.59	3.07 ± 0.49	−0.88 (−2.96 to 1.19)	Paired *t*-test	Cohen’s d = −0.39	0.337
Insulin (mIU/L)	10.34 ± 10.57	5.54 ± 1.61	−4.80	Wilcoxon signed-rank test	—	0.375
HOMA-IR	3.74 ± 3.22	1.74 ± 0.39	−2.00	Wilcoxon signed-rank test	—	0.156
Total cholesterol (mg/dL)	161.00 ± 39.29	164.43 ± 38.52	3.43 (−21.28 to 28.14)	Paired *t*-test	Cohen’s d = 0.13	0.746
Triglyceride (mg/dL)	173.00 ± 107.00	150.86 ± 42.09	−22.14 (−93.44 to 49.16)	Paired *t*-test	Cohen’s d = −0.29	0.476
HDL-cholesterol (mg/dL)	45.86 ± 17.02	44.57 ± 14.35	−1.29	Wilcoxon signed-rank test	—	0.531
Total cholesterol/HDL ratio	3.70 ± 0.73	3.83 ± 0.49	0.13 (−0.24 to 0.49)	Paired *t*-test	Cohen’s d = 0.33	0.422
LDL-cholesterol (mg/dL)	93.71 ± 26.35	97.29 ± 23.35	3.57 (−13.24 to 20.39)	Paired *t*-test	Cohen’s d = 0.20	0.622
LDL/HDL ratio	2.17 ± 0.62	2.27 ± 0.42	0.10 (−0.21 to 0.41)	Paired *t*-test	Cohen’s d = 0.30	0.455
CRP (mg/L)	1.30 (1.20, 1.43)	2.60 (1.30, 4.06)	1.74 (−0.47 to 3.96)	Wilcoxon signed-rank test	Rank-biserial correlation = 0.64	0.156

Data are presented as mean ± SD unless otherwise indicated. CRP is presented as median (Q1, Q3) because of its non-normal distribution. Abbreviations: Glucose AC, fasting blood glucose; HOMA-IR, homeostasis model assessment of insulin resistance; HDL, high-density lipoprotein; LDL, low-density lipoprotein; CRP, C-reactive protein.

## Data Availability

The data presented in this study are available from the corresponding author upon reasonable request.

## Abbreviations

The following abbreviations are used in this manuscript:

T2DM	Type 2 diabetes mellitus
PBM	Photobiomodulation
CNS	Central nervous system
ANS	Autonomic nervous system
ATP	Adenosine triphosphate
CRP	C-reactive protein

## References

[1] International Diabetes Federation (2021). IDF Diabetes Atlas.

[2] Schwartz M.W., Woods S.C. (2000). Central nervous system control of food intake. Nature.

[3] Morton G.J., Meek T.H., Schwartz M.W. (2014). Neurobiology of food intake in health and disease. Nat. Rev. Neurosci..

[4] Myers M.G., Olson D.P. (2012). Central nervous system control of metabolism. Nature.

[5] Schwartz M.W., Porte D. (2005). Diabetes, obesity, and the brain. Science.

[6] Pocai A. (2012). Unraveling oxyntomodulin, GLP1’s enigmatic brother. J. Endocrinol..

[7] Berthoud H.R., Münzberg H. (2011). The lateral hypothalamus as integrator of metabolic and environmental needs. Physiol. Behav..

[8] Thayer J.F., Lane R.D. (2009). Claude Bernard and the heart–brain connection: Further elaboration of a model of neurovisceral integration. Neurosci. Biobehav. Rev..

[9] Esler M., Lambert G., Schlaich M. (2010). Chronic activation of the sympathetic nervous system is the dominant contributor to systemic hypertension. J. Appl. Physiol..

[10] Karu T.I. (2008). Mitochondrial signaling in mammalian cells activated by red and near-IR radiation. Photochem. Photobiol..

[11] Hamblin M.R. (2017). Mechanisms and applications of the anti-inflammatory effects of photobiomodulation. AIMS Biophys..

[12] Chung H., Dai T., Sharma S.K., Huang Y.Y., Carroll J.D., Hamblin M.R. (2012). The nuts and bolts of low-level laser (light) therapy. Ann. Biomed. Eng..

[13] de Freitas L.F., Hamblin M.R. (2016). Proposed mechanisms of photobiomodulation or low-level light therapy. IEEE J. Sel. Top. Quantum Electron..

[14] Hamblin M.R. (2016). Shining light on the head: Photobiomodulation for brain disorders. BBA Clin..

[15] Salehpour F., Hamblin M.R. (2018). Brain photobiomodulation therapy: A narrative review. Rev. Neurosci..

[16] Naeser M.A., Hamblin M.R. (2015). Traumatic brain injury: A major medical problem that could be treated using transcranial red/near-infrared LED photobiomodulation. Photomed. Laser Surg..

[17] Barrett D.W., Gonzalez-Lima F. (2013). Transcranial infrared laser stimulation produces beneficial cognitive and emotional effects in humans. Neuroscience.

[18] Cassano P., Petrie S.R., Mischoulon D., Cusin C., Katnani H., Yeung A., De Taboada L., Archibald A., Bui E., Baer L. (2018). Transcranial Photobiomodulation for the Treatment of Major Depressive Disorder. The ELATED-2 Pilot Trial. Photomed. Laser Surg..

[19] Cassano P., Petrie S.R., Mischoulon D., Cusin C., Katnani H., Nierenberg A.A., Yeung A., De Taboada L., Archibald A., Bui E. (2019). Transcranial photobiomodulation for the treatment of major depressive disorder. Photobiomodul. Photomed. Laser Surg..

[20] Cassano P., Petrie S.R., Hamblin M.R., Henderson T.A., Iosifescu D.V. (2016). Review of transcranial photobiomodulation for major depressive disorder. Neurophotonics.

[21] Salehpour F., Mahmoudi J., Kamari F., Sadigh-Eteghad S., Rasta S.H., Hamblin M.R. (2018). Brain Photobiomodulation Therapy: A Narrative Review. Mol. Neurobiol..

[22] Gagnier J.J., Kienle G., Altman D.G., Moher D., Sox H., Riley D., CARE Group (2014). The CARE Guidelines: Consensus-Based Clinical Case Reporting Guideline Development. J. Clin. Epidemiol..

[23] World Medical Association (2013). World Medical Association Declaration of Helsinki: Ethical Principles for Medical Research Involving Human Subjects. JAMA.

[24] American Diabetes Association (2025). Classification and diagnosis of diabetes: Standards of Care in Diabetes—2025. Diabetes Care.

[25] Cheng Z.L. (2020). Craniosacral Therapy: Skull Volume (頭薦療法—頭骨篇).

